# Molecular Biomarkers for the Diagnosis of Primary Vitreoretinal Lymphoma

**DOI:** 10.3390/ijms12095684

**Published:** 2011-09-05

**Authors:** Yujuan Wang, Defen Shen, Vinson M. Wang, H. Nida Sen, Chi-Chao Chan

**Affiliations:** 1 Immunopathology Section, Laboratory of Immunology, National Eye Institute, National Institutes of Health, Bethesda, MD 20892, USA; E-Mails: yujuan.wang@nih.gov (Y.W.); shend@nei.nih.gov (D.S.); vinson.wang@nih.gov (V.M.W.); 2 Zhongshan Ophthalmic Center, Sun Yat-sen University, Guangzhou 510060, China; 3 Clinical Immunology Section, Laboratory of Immunology, National Eye Institute, National Institutes of Health, Bethesda, MD 20892, USA; E-Mail: senh@nei.nih.gov

**Keywords:** primary vireoretinal lymphoma, biomarker, immunoglobulin heavy chain, T-cell receptor, gene arrangement, microdissection, polymerase chain reaction

## Abstract

Primary vitreoretinal lymphoma (PVRL) or primary intraocular lymphoma, a subtype of primary central nervous system lymphoma, often masquerades as uveitis. The diagnosis of PVRL requires identification of lymphoma cells inside the eye, which is often challenging due to the frequent necrosis and admixing of PVRL cells with reactive lymphocytes. Therefore, detection of *immunoglobulin heavy chain* (*IgH*) and *T-cell receptor* (*TCR*) gene rearrangements provide molecular diagnosis of B- and T-cell lymphoma, respectively. We retrospectively evaluated 208 cases with a clinical diagnosis of masquerade syndrome from 1998 to 2010. In 200 cases with molecular analyses using microdissection and polymerase chain reaction, we found that 110 cases had *IgH* gene rearrangement, 5 cases had *TCR* gene rearrangement, and 85 cases were negative for these two gene arrangements. The molecular data corroborated the cytopathological diagnoses of PVRL and uveitis in the majority of cases. Cytokine above the detected levels in the specimens were also measured in 80 of the 208 cases. A ratio of vitreous IL-10 to IL-6 greater than 1, suggesting PVRL, was found in 56/80 cases; 53/56 had the correct diagnosis. A ratio less than 1, suggesting uveitis, was found in 24/80 cases; 17/24 correctly confirmed the diagnosis. Moreover, the molecular data corresponded well with the clinical course of the diseases. The sensitivity and specificity of these molecular biomarkers for the diagnosis of PVRL are higher than 95%.

## 1. Introduction

Primary vitreoretinal lymphoma (PVRL), also known as primary intraocular lymphoma, is a subset of primary central nervous system lymphoma (PCNSL) [1]. PVRL initially involves the vitreous, retina, subretinal space, and optic nerve with or without simultaneous CNS involvement [2,3]. The majority of PVRL is extranodal, non-Hodgkin, diffuse large B-cell lymphoma (DLBCL) [4]. Although rare, T-cell lymphoma with intraocular involvement has also been reported, usually with poor prognosis [5–7].

**Figure 1:**
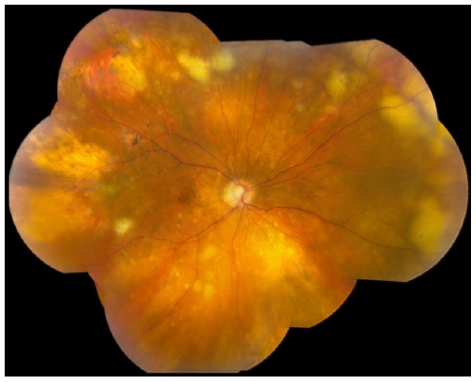
Fundoscopy of a patient with primary vitreoretinal lymphoma (PVRL) showing multiple yellowish subretinal lesions and small areas of retinal necrosis.

Clinically, PVRL often masquerades as uveitis, which is most commonly characterized by vitritis, presence of cells in the anterior chamber, and subretinal yellow infiltrates (Figure 1) [8–10]. Clinical manifestations, fundoscopic and ocular imaging (autofluorescence, fluorescein angiography and optic coherence tomography) often provide characteristic features of PVRL. Diagnosis of PVRL requires pathologic identification of atypical lymphoma cells from the vitreous or retina. Cerebrospinal fluid (CSF), aqueous humor, subretinal fluid, and intraocular tissues (such as chorioretinal tissue or iris) are also used for pathology [11]. However, the cytopathologic diagnosis is challenging because of the fragility and paucity of the atypical lymphoma cells in the vitreous [12]. Moreover, atypical lymphoma cells in the vitreous often admix with reactive lymphocytes, necrotic cells and debris, adding to the difficulty of diagnosis. Additionally, pathologic diagnosis can only determine the presence of tumor cells instead of identifying them as B- or T-cell lymphoma.

Monoclonal rearrangements of *immunoglobulin heavy chain* (*IgH*) and *T-cell receptor* (*TCR*) genes can be detected and these molecular techniques provide a reliable biomarker for lymphoma cells. The *IgH* gene in B lymphocytes is comprised of three highly variable complementarity determining regions (CDR), separated by four relatively conserved framework regions (FR) [13–15]. Among them, CDR3 is the most variable region of the rearranged *IgH* gene and can be detected by several primers such as *FR2A*, *FR3A*, and *CDR3* [8,16]. Similarly, *TCR* gene rearrangement, most commonly *TCR gamma* gene rearrangement, serves as a molecular marker of clonal expansion of T-cell lymphoma [5,17]. *TCR* gene rearrangement can be detected by primers such as *TCR-gamma* and *TCR-CDR3* [18,19]. By using microdissection to obtain at least 15 atypical lymphoid cells and performing polymerase chain reaction (PCR), specific gene rearrangements can be detected in these specimens from lymphoma patients [20]. Thus, molecular analysis provides a useful adjunct to the diagnosis as well as classification of PVRL.

Interleukin-10 (IL-10) is regarded as a growth and differentiation factor for B lymphocytes [21]. The level of IL-10 is increased in the vitreous and CSF of patients with PCNSL [22–24]. IL-6, which is produced by a wide variety of cell types including inflammatory cells, is a marker of inflammatory diseases [25]. IL-6 is simultaneously measured in the same specimen in order to internally control for the dilution of the vitreous during sample collection. Previous studies have found that both elevated IL-10 levels and a high IL-10 to IL-6 ratio in the vitreous are indicative of PVRL in many patients [22,23,26]. An IL-10 to IL-6 ratio greater than 1 is the most useful predictor of lymphoma. On the other hand, an IL-10 to IL-6 ratio of less than 1 suggests the presence of uveitis. The IL-10 to IL-6 ratio can be influenced by many factors, such as previous corticosteroids or immunosuppressive treatments and certain systemic diseases that may influence various cytokine productions, which limits its diagnostic power. However, significantly elevated IL-10 levels (>400 pg/mL in the undiluted vitreous) alone may also raise suspicion of intraocular lymphoma [24].

This study focuses on the molecular analysis of *IgH* and *TCR* gene arrangements as the biomarker of the B- and T-cell PVRL for the diagnosis and classification of PVRL. The diagnostic value (including sensitivity, specificity, positive and negative predictive value, and efficiency) of molecular analysis, cytopathologic findings, and the IL-10 to IL-6 ratio are compared in patients with the clinical diagnosis of ocular masquerade syndrome that are highly suspected of PVRL. Our findings demonstrate that molecular results correlate well with the clinical course of PVRL and contribute to the clinical diagnosis and classification of PVRL.

## 2. Results and Discussion

### 2.1. Patient Demographics

A total of 227 specimens from 214 patients clinically diagnosed with masquerade syndrome were identified from 1998 to 2010 at the National Eye Institute, National Institutes of Health. All the patients involved are highly suspected of PVRL. Of these, there was one specimen per patient for 203 patients and multiple specimens for 11 patients. Total pathologic specimens comprised of 175 vitreous, 11 aqueous, 21 CSF, 5 subretinal fluid, 4 iris, 7 retina/chorioretina, 3 brain, and 1 orbital tissue (Table 1).

### 2.2. Clinical Findings

Among 214 patients, PVRL was confirmed in 119 patients, and uveitis in 89 patients. In 6 patients, a definitive diagnosis could not be reached due to insufficient specimen for cytological, cytokine and/or molecular analysis (Table 2). Therefore, 208 patients with definitive diagnoses were included in the final analysis.

The average age showed no statistical difference between PVRL and uveitis patients (65.2 ± 12.4 *vs*. 65.8 ± 13.9, *P* = 0.60, Table 2). The gender distribution was not significantly different between PVRL and uveitic patients (1:1.2 *vs*. 1:1.8, *P* = 0.18, Table 2).

The majority of patients (81.7%) did not have other systemic malignancies. Of the 38 patients who had systemic malignancies, most had lymphoma; only one patient had PVRL and systemic non-hematological tumor (Table 3). Interestingly, 13 uveitis patients also had hematological malignancies (11 lymphoma and 2 leukemia).

### 2.3. Molecular Analysis

**Figure 2:**
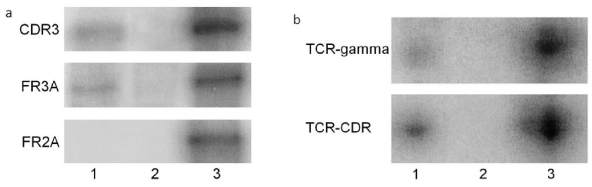
PCR of gene rearrangements in two patients with PVRL: (**a**) *IgH* gene rearrangement of B-cell PVRL was detected using primer pairs of CDR3 and FR3A but not FR2A. (**b**) *TCR* gene rearrangement of T-cell PVRL was detected using primer pairs of TCR-gamma and TCR-CDR (Lane 1, vitreous specimen; Lane 2, negative control; Lane 3, positive control).

Microdissection of the atypical lymphoid cells and PCR were performed for 200 of the 208 patients (Table 4). Among the 200 patients, 114 were diagnosed with PVRL and showed *IgH* (109 cases with B-cell lymphoma, Figure 2a) or *TCR* (5 cases with T-cell lymphoma, Figure 2b) gene rearrangements. In contrast, 85 of the 86 uveitic patients showed negative *IgH* or *TCR* gene rearrangements; only one uveitic patient had positive *IgH* gene rearrangement.

### 2.4. Cytological and Pathological Findings

**Figure 3:**
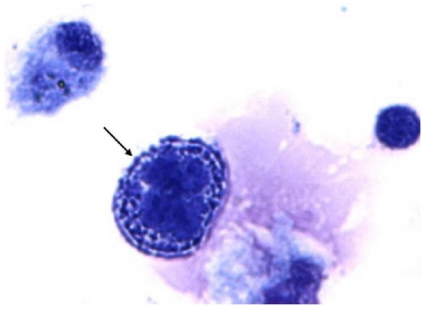
Cytology of a patient with PVRL showing a large atypical lymphoid cell (arrow) with large nuclei, prominent nucleoli, and scanty basophilic cytoplasm in the vitreous specimen (Giemsa, original magnification, ×640).

Among the 208 patients who had defined diagnoses, 205 had specimens for cytological analysis to identify atypical lymphoid cells (Figure 3). Twenty-three of 205 patients’ specimens did not have identifiable cells due to poor morphology. A total of 182 cases were included for cytological analysis (Table 5).

### 2.5. Cytokine Analysis

IL-10 and IL-6 analysis were performed in 118 of the 208 patients. Thirty-eight patients had below detectable levels of both IL-10 and IL-6; therefore, the IL-10 to IL-6 ratio could not be calculated in these patients. The IL-10 to IL-6 ratio was available for 80 patients and the results are listed in Table 6. The ratio was less than 1 in 7 PVRL patients, including 3 of the 5 T-cell lymphoma cases.

All patients were treated based on the molecular diagnosis accordingly. Clinical follow-up on these patients also confirmed the diagnosis. Overall, the molecular technique has the highest diagnostic value in PVRL among the three methods analyzed in our study (Table 7). Cytology has higher specificity and positive predictive value compared with cytokine analysis in PVRL patients. However, cytokine analysis has higher sensitivity and negative predicative value.

### 2.6. Discussion

PVRL is a potentially fatal malignant disease and accurate diagnosis is of critical importance for prompt and appropriate intervention. The gold standard for PVRL diagnosis is detection of lymphoma cells in the retina and/or vitreous. Additionally, clinical manifestations and examinations are important for the diagnosis of PVRL. This study suggests that the molecular analysis of *IgH* and *TCR* gene rearrangements using microdissection and PCR technique has the highest sensitivity, specificity, predictive value, and efficiency for the diagnosis of PVRL, when compared with morphological identification of atypical lymphoid cells or IL-10 to IL-6 ratio analysis. Moreover, detection of *IgH* or *TCR* gene rearrangements in the microdissected atypical lymphoid cells greatly facilitates the diagnosis and classification of PVRL. The molecular analysis correlates well with the clinical diagnosis of PVRL and can be used as reliable biomarkers for the diagnosis and classification of PVRL.

PVRL usually masquerades as uveitis; thus, identification of lymphoma cells in the eye is required for the diagnosis of PVRL. Typical lymphoma cells are characterized by large nuclei, prominent nucleoli, and scanty basophilic cytoplasm [27,28]. These atypical lymphoid cells are 2–4 times the size of normal, naive lymphocytes and may have irregular nuclear contours, a fine to coarse chromatin pattern and scanty basophilic cytoplasm [29]. Although PVRL cells have characteristic features, they are very fragile and easily degenerate in the specimen and even in the eye [8,12]. If the specimen is not promptly transported and/or carefully processed, most PVRL cells will show degeneration or may even become necrotic. In this study, 12% of the specimens did not have identifiable cells due to poor morphology, resulting in no diagnosis based on cytology alone.

Although cytological analysis is critical for identifying tumor cells in PVRL, further classification of the cell origin of lymphoma cells must rely on immunophenotyping using techniques such as immunohistochemistry or flow cytometry. Immunohistochemistry staining of the B cell marker (e.g., CD19, CD20, lambda, and kappa) and T cell marker (e.g., CD3) help differentiate the monoclonality of lymphoma cells. Other studies have reported that flow cytometric immunophenotyping is a useful alternative to conventional diagnostic techniques. It refines cytological diagnosis and helps in the classification of ocular lymphoma [30,31]. However, flow cytometric immunophenotyping requires relatively larger quantities of material and ocular specimens are often limited to very small amounts [31]. Thus, the paucity and fragility of the lymphoma cells in the eye restrict the application of immunophenotyping analysis for PVRL diagnosis and classification.

PVRL cells show monoclonal rearrangement of *IgH* genes in B-cell lymphoma and *TCR* genes in T-cell lymphoma. This feature favors the molecular diagnosis and classification of lymphoma using microdissection to procure the atypical lymphoid cells and PCR to detect gene rearrangements in these cells. The antigen-binding sites of IgH and TCR molecules are encoded by sets of gene regions that include variable (V), diversity (D), and jointing (J) gene regions. The variable region undergoes gene rearrangements that increase the diversity of the immune receptor repertoire [17,32]. Among them, *CDR3* in the V(D)J region of the *IgH* gene and *TCR gamma* in *TCR* gene are regarded as the most common sites of gene arrangement [5,8,16,17]. Because lymphoma usually derives from a single B or T cell, the tumor cell has the features of monoclonality. Thus, gene rearrangements of *IgH* and *TCR* in lymphoma cells serve as reliable biomarkers for the diagnosis and classification of PVRL. In this study, 114 cases of B-cell lymphoma are identified by molecular analysis using primers *FR2A*, *FR3A*, and/or *CDR3* that cover the *CDR3* region of the *IgH* gene in B-cell lymphoma. Five cases of T-cell PVRL are confirmed by the positive *TCR* gene arrangement using primer *TCR-gamma* and/or *TCR-CDR3* in T-cell PVRL. Only one uveitis case showed *IgH* gene rearrangement in the *CDR3* region. No atypical lymphoid cells were found and a proinflammatory cytokine profile was measured in this specimen; therefore, the molecular data alone could not support a diagnosis of lymphoid neoplasm for this patient. The patient responded to anti-inflammatory therapy eventually. Though rare, molecular analysis could yield false positive results.

Because PVRL cells often admix with many reactive lymphocytes and necrotic cells, microdissection of at least 15 atypical lymphoid cells in individual specimens likely guarantees the purity of the cell source for PCR, which greatly improves the accuracy of PVRL diagnosis via molecular analysis. By using microdissection and PCR, the sensitivity and specificity was nearly 100% in patients with PVRL in our study. In comparison, others reported molecular diagnosis rates of approximately 60% [33,34] and specificity around 0.64 in smaller case series [35]. In those molecular studies, however, microdissection was not performed.

Vitreous biopsy remains the mainstay of PVRL diagnosis. Clinically, vitreous fluids are obtained by vitreous aspiration or pars plana vitrectomy. Our results showed that the majority of submitted specimens (77%) were vitreous samples. Vitreous and aqueous fluids often have sparsely distributed tumor cells admixed with many reactive lymphocytes and debris, which make it difficult for pathologists to differentiate tumor cells from inflammatory cellular contents. Moreover, lymphoma cells tend to have poor morphology due to prior corticosteroid treatment, mechanical damage during vitrectomy and/or prolonged processing procedure [11,36,37]. All of these reasons make PVRL diagnosis challenging. Aqueous is anatomically farther away from the lymphoma lesions than the vitreous simply because PVRL presents in the vitreous and retina. Although fewer PVRL cells are present in the aqueous than in the vitreous, lymphoma cells were found in 2 of the 4 PVRL cases with aqueous specimens. Of the same 4 PVRL cases, 3 had elevated IL-10 levels, which facilitated the final diagnosis of PVRL. Cassoux *et al.* also reported that cytokine measurements of the aqueous could be used as a good screening method for PVRL diagnosis [24]. Other intraocular specimens, such as retina/chorioretina and subretinal fluids, are usually chosen when PVRL cells are absent in the vitreous samples [38–41]. In general, PVRL cells are easier to detect in the retina and subretinal space, where they initially reside.

An IL-10 to IL-6 ratio greater than 1 in ocular fluid is highly suggestive of PVRL [26]. However, IL-10 secretion is affected by not only activated and malignant B cells, but also by other cells, such as Th1 and Th2 cells [42,43], and by many other factors, such as IL-12 levels [44]. In our study, 3 uveitic specimens (2 vitreous and 1 aqueous) showed elevated vitreous IL-10 levels and an IL-10 to IL-6 ratio greater than 1. Akpek *et al*. [45] also reported that the IL-10 to IL-6 ratio was greater than 1 in 8 of 13 patients with non-neoplastic uveitis. In contrast, 7 (11.7%) PVRL cases showed a vitreous IL-10 to IL-6 ratio less than 1 in our study. Three of these were of T-cell origin and the low IL-10 level was compatible with the diagnosis of T-cell lymphoma [21]. Low IL-10 to IL-6 levels in B-cell lymphomas have also been reported in other studies [45,46]. This change may occur in patients who were in the early stage of lymphoma or in patients who have been previously treated with corticosteroids or immunosuppressive agents that influence the cytokine profile. Although an IL-10 to IL-6 ratio greater than 1 in the vitreous is suggestive of PVRL, this ratio cannot be used as the sole biomarker for PVRL diagnosis. However, cytokine analysis showed higher sensitivity and negative predicative value in this study, suggesting that it may serve as a useful adjunctive tool for screening patients suspected of PVRL.

## 3. Experimental Section

The medical records of 214 patients clinically diagnosed with masquerade syndrome and highly suspected of PVRL were reviewed retrospectively. All cases were diagnosed at the National Eye Institute from 1998 to 2010. The study was approved by the National Eye Institute Institutional Review Board for human subjects and informed consent was obtained from all patients.

The protocol for specimen processing has been outlined in related studies [8,41]. Briefly, the aqueous, vitreous, subretinal fluid, or CSF obtained from surgeries was processed. The fluid was centrifuged at 1000 rpm. The supernatant was collected and IL-10 and IL-6 levels were measured using enzyme-linked immunosorbent assay. The sediment in the original tube was then resuspended and cytocentrifuged at 1000 rpm using CytoPro 7620 cytocentrifuge (Wescor Inc., UT, USA). The cytospin slides were stained with Giemsa stain for cytology and molecular analyses. The biopsy tissue specimens (including iris, retina/chorioretina, brain, and orbital tissue) were embedded either in paraffin or OCT (Sakura Finetek USA Inc., CA, USA). These slides were routinely stained with hematoxylin and eosin for histology and molecular analyses.

A minimum of 15 atypical cells were microdissected from the cytospin slides and digested with Proteinase K buffer. PCR and gel electrophoresis were used to detect the monoclonality of malignant B and T cells. The *CDR3* of the *IgH* gene rearrangement in B-cell PVRL was detected by the ^32^P labeled primer pairs of *FR2A* (sense 5′-TGG RTC CGM CAG SCV YCN GG-3′ and antisense 5′-GGA TGG TAC CAA GCT TTG AGG AGA CGG TGA CCA-3′), *FR3A* (sense 5′-ACA CGG CYS TGT ATT ACT GT-3′ and antisense 5′-GGA TGG TAC CAA GCT TTG AGG AGA CGG TGA CCA-3′) and *CDR3* (sense 5′-CCG GRA ARR GTC TGG AGT GG-3′ and antisense 5′-ATC CTG AGG AGA CGG TGA CC-3′). *TCR* gene rearrangement in the T-cell PVRL was detected by the ^32^P labeled primer pairs of *TCR-gamma* (sense 5′-AGG GAT GTG TTG GAA TCA GG-3′ and antisense 5′-CGT CGA CAA CAA GTG TTG TTC CAC-3′) and *TCR-CDR3* (sense 5′-GAA AGG AAT CTG GCA TTC CGT CAG-3′ and antisense 5′-GAA GTT ACT ATG AGC YTA GTC CCT T-3′) [19]. After gel electrophoresis and autoradiography overnight, the PCR products are visible by using molecular imager (Bio-Rad Laboratories, CA, USA).

The student’s *t*-test was used to calculate numerical data and the chi-square test was used for categorical data. The analysis was performed using SPSS version 17.0 (SPSS Inc., IL, USA). *P* values less than 0.05 are considered statistically significant. The diagnostic value was recorded by sensitivity, specificity, positive and negative predictive value, and efficiency.

## 4. Conclusions

This study indicates that *IgH* and *TCR* gene rearrangements are reliable biomarkers for B- and T-cell PVRL, respectively. Using the molecular technique PCR to detect these gene rearrangements in microdissected atypical lymphoid cells has a higher diagnostic value than those from cytology and cytokine analyses. Although identification of intraocular tumor cells remains the gold standard for PVRL diagnosis, molecular analysis of DNA obtained by PCR provides valuable supporting diagnostic data, especially in specimens with a limited number of cells and/or those with poor morphology. The accuracy of the molecular analysis depends largely on the combination of microdissection and PCR, which allows for selection of individual atypical lymphoid cells and ensures detection of the monoclonal characteristics of lymphoma cells.

Through identification of specific *IgH* and *TCR* gene arrangements, molecular analysis contributes to further classification and confirmation of PVRL with only a small number of lymphoma cells, whereas routine cytological and cytokine analyses may not be able to provide such information from small specimens. Because B- and T-cell lymphomas respond differently to certain therapeutic agents such as Rituximab (monoclonal anti-CD20 antibody), which is effective in B-cell lymphoma [47], oncologists can choose the most effective targeted treatment for each individual PVRL patient based on specific lymphoid cell origins.

## Figures and Tables

**Table 1:** Specimen submission in patients with masquerade syndrome.

Specimensper Patient	Number of Patients	Specimen Type (*n* = patients) *	Number of Specimens
1	203	vitreous (164), aqueous (10), CSF (14), subretinal fluid (2), iris (3), retina/chorioretina (6), brain (3), orbital tissue (1)	203
2	9	vitreous + iris (1), vitreous+chorioretina (1), vitreous + CSF (5), vitreous+subretinal fluid (2)	18
3	2	vitreous + CSF + subretinal fluid (1), vitreous + CSF + aqueous (1)	6

Total	214	227	

* The number of patients who submitted each specimen type is indicated in parentheses.

**Table 2:** Demographics in patients of masquerade syndrome.

	PVRL	Uveitis	Total
Number	119 *	89	208
Age (mean ± SD)	65.2 ± 12.4	65.8 ± 13.9	65.5 ± 13.1
Gender (male:female)	1:1.2	1:1.8	1:1.4

* Five of 119 patients were identified with T-cell lymphoma.

**Table 3:** Distribution of systemic malignancies in patients with masquerade syndrome.

	with Systemic Malignancy	without Systemic Malignancy		
		Lymphoma	Leukemia	Other Malignancy
PVRL	67	20	2	1
PVRL + PCNSL	27	2	0	0
Uveitis	72	11	2	0
Uveitis + PCNSL	4	0	0	0

Total	170	33	4	1

**Table 4:** Molecular analysis.

*IgH*/*TCR* Gene Rearrangements	PVRL	Uveitis	Total
Positive	114	1	115
Negative	0	85	85
Total	114	86	200

Sensitivity = 1.0			
Specificity = 0.99			
Positive predictive value = 0.99			
Negative predictive value = 1.0			
Test efficiency = 0.995			

**Table 5:** Cytological and pathological analysis.

Atypical Lymphoid Cells	PVRL	Uveitis	Total
Present	81	1 *	82
Absent	19 †	81	100
Total	100	82	182

Sensitivity = 0.81			
Specificity = 0.99			
Positive predictive value = 0.99			
Negative predictive value = 0.81			
Test efficiency = 0.890			

* There are less than 15 atypical lymphoid cells identified in the specimen and the majority of the lymphocytes are CD3 positive T cells in this case.

† The specimens contained atypical lymphoid cells with poor morphology, which are highly suspected of lymphoma cells.

**Table 6:** Cytokine analysis.

IL-10 to IL-6 Ratio	PVRL	Uveitis	Total
>1	53	3	56
<1	7	17	24
Total	60	20	80

Sensitivity = 0.88			
Specificity = 0.85			
Positive predictive value = 0.95			
Negative predictive value = 0.71			
Test efficiency = 0.875			

**Table 7:** Comparison of the diagnostic value in molecular, cytological, and cytokine analysis.

Diagnostic Method	Sensitivity	Specificity	Positive Predictive Value	Negative Predictive Value	Test Efficiency
*IgH*/*TCR* gene rearrangements	1	0.99	0.99	1	0.995
Cytology	0.81	0.99	0.99	0.81	0.890
IL-10/IL-6 ratio	0.88	0.85	0.95	0.71	0.875
